# Naval Surgeons

**Published:** 1848-01

**Authors:** 


*Naval Surgeons.*—The Board of Naval Surgeons, which has been in session at the Naval Asylum since the 11th of October, has adjourned under order of the Navy Department, to meet again on the 10th of January next. Candidates will have time to prepare for examination. The following candidates for admission have been examined by the Board recently in session, found qualified, and commissioned as assistant surgeons to rank in the following order:—1. William Lowber. 2. D. Warren Brickell. 3. Geo. H. Howell. 4. D. P. Phillips. 5. Ashton Miles. 6. Phineas S. Horwitz.

In connection with this subject, the following communication, which we find in the Washington Union, possesses peculiar interest:—

*Want of Medical Officers in the Navy.*—Within a few days intelligence has been received of the death of three of the most able and efficient naval surgeons, from yellow fever, at the hospital on the island of Salmadina near Vera Cruz. The number of sick in the squadron was still large, and an additional number of medical officers are required upon that as well as upon other stations, while the present number, limited by law, is inadequate to the wants of the service; and *every annual report from the Secretary of the Navy, for the last four years, has complained of the paucity of medical officers, and asked for an increase of that corps.*

Three years ago, the gentleman at present at the head of the department, and who is so familiar with all the wants of the service, urgently asking for an increase, remarked, "that several of the most industrious and efficient members of the corps had resigned in consequence of the continued arduous duties unavoidably imposed upon them, and their being immediately reordered to sea after long and protracted cruizing." This state of things continues to exist, and Congress alone can apply the remedy.

The government is most assuredly bound to supply with medical aid those brave officers and men, who not only brave death at the cannon's mouth but also live amidst disease and pestilence in the most insalubrious climates on the face of the globe.

On reference to the Navy Registers, as far back as our war in 1814, the cause of this disproportion of medical officers becomes obvious. While all the other grades in the navy have been tripled and quadrupled, while our ships have been so much increased in numbers, and the cruising stations augmented by the addition of squadrons on the coast of Africa and in the East Indies; whilst many of the senior surgeons have been rendered incapable of active service, from advanced age or from impaired health, the corps of medical officers have received little or no addition, while their duties have been so much augmented.

The following table, taken from the official Navy Register, will exhibit the number of officers of each grade in 1815 and 1847, with the increase:

| | 1815. | 1847. | Increase. |
|---|---|---|---|
| Captains, - - - | 32 | 64 | 32 |
| Commanders, - - - | 18 | 97 | 79 |
| Lieutenants, - - - | 140 | 324 | 184 |
| Surgeons, - - - | 48 | 69 | 21 |
| Assistant Surgeons, - - | 72 | 67 | |

The above table shows the increase of all classes of officers, save the medical corps, with the increase of the navy and the rapid growth of the whole country. At present the number of surgeons in the navy is inadequate to the performance of the duties.

It is earnestly hoped that the next Congress will act promptly and speedily upon this matter, and make such an increase of the corps as the service demands, and the Secretary shall recommend. They will thereby not only perform an act of duty, but one of HUMANITY.

